# Prevalence, correlates, and trends of intimate partner violence against women in Sierra Leone: findings from 2013 and 2019 demographic and health surveys

**DOI:** 10.3389/fpubh.2023.1227165

**Published:** 2023-10-18

**Authors:** Masood Ali Shaikh

**Affiliations:** Independent Researcher, Karachi, Pakistan

**Keywords:** intimate partner violence, trend, disparity, women, Sierra Leone

## Abstract

**Background:**

Intimate partner violence (IPV) is a globally pervasive public health and medical problem in addition to being a serious violation of women’s rights. The two-fold objectives of this study were to compute the lifetime prevalence and correlates of IPV perpetrated by current/former husbands/partners of ever-married women aged 15–49 years using the nationally representative Sierra Leone Demographic and Health Survey (DHS) conducted in 2019; ethnicity-based levels and trends of IPV were also computed using the data from the DHS conducted in 2013 along with the 2019 DHS.

**Methods:**

Twelve IPV correlates pertaining to socio-demographic, attitudinal, and experiential attributes were analyzed using logistic regression models for bivariate and multivariable analysis. IPV was computed as a composite variable comprising of physical, emotional, and/or sexual IPV.

**Results:**

Lifetime prevalence of experiencing any kind of IPV was a staggering 60.81%, whereas emotional, physical, and sexual IPV prevalence were reported by 45.90%, 49.81%, and 8.14% of the respondents, respectively. No ethnicity reported a statistically significant decrease in any type of IPV during the intervening period between the two surveys. Five out of nine ethnicities reported a statistically significant increase in emotional IPV, while few ethnicities reported a statistically significant increase in one or more types of IPV.

**Conclusion:**

The high lifetime prevalence of IPV is alarming and points to the need for targeted health promotion campaigns to reverse the tide of IPV in Sierra Leone, including focusing on changes in cultural and ethnicity-based norms and mores to ensure women’s human rights are respected and upheld.

## Introduction

“Love hurts” is an astute observation and the title of a 1960 song written by the American songwriter Boudleaux Bryant. For a sizable proportion of ever-married women, intimacy with men comes at the cost of violence and pain. Intimate partner violence (IPV) is a global public health and medical problem that is also a serious human rights violation. IPV can manifest as either physical, sexual, and/or emotional violence, which is a health problem that transcends cultures and geographies across millennia (1). It is defined as “any behavior within an intimate relationship that causes physical, psychological, or sexual harm to those in the relationship” (2). The prevalence estimate of lifetime physical and/or sexual IPV for ever-married/partnered women aged 15–49 in the WHO Africa Region is reported at 33%, contrasting with the global estimate of 27% (3).

The nationally and sub-nationally representative Demographic and Health Surveys (DHSs) are cross-sectional household surveys conducted in countries by various national health and statistics ministries/departments in collaboration with the United States Agency for International Development (USAID). DHSs have been conducted in over 90 countries globally, including African countries (4). Several studies using DHS data have identified a host of correlates for IPV; these include demographic and social factors, cultural attitudes, and practices.

Two meta-analyses using DHS data identified residency in rural areas and lower educational attainment in women as correlates of intimate partner violence. A meta-analysis based on 44 DHSs from 29 Sub-Saharan African (SSA) countries found associations between higher levels of IPV with low educational attainment in women and rural residency status (5), while another meta-analysis based on DHSs conducted in 25 SSA countries found similar associations of poor living conditions and IPV with rural residency and low educational attainment in women (6). A meta-analysis based on 36 prospective longitudinal studies found parental educational attainment below the stage of high school and unplanned pregnancy as risk factors for IPV, while older age and being married were found to be protective factors against experiencing IPV (7). Another meta-analysis based on 25 studies from low- and middle-income countries found higher IPV prevalence in infertile women (8). Using DHS data from various countries, several studies have identified a surfeit of IPV correlates that include age, educational attainment, urban/rural residency status, employment status, household income, partner’s use of alcohol, acceptance/justification of violence, role in decision-making, partner’s controlling behavior, and knowledge of or exposure to parental physical violence (9–26).

The Republic of Sierra Leone forms the southwest coast of West Africa, with an estimated population of eight million in the year 2019 (27). A limited number of studies have been published on the prevalence and correlates of intimate partner violence from Sierra Leone, including studies using DHS data. In a study using focus groups and individual interviews, women from Sierra Leone and Liberia identified their financial dependence on men, culturally defined gender expectations, and wars leading to amplified use of violence as correlates of IPV (28). Using DHS data from 2011–2015 surveys conducted in sub-Saharan Africa countries, including Sierra Leone, controlling behavior of husbands/partners was identified as a strong correlate of IPV (29). Another study using DHS data from DHSs conducted between 2012 and 2020 in countries including Sierra Leone reported a strong association between exposure to interparental violence and IPV (30).

The objectives of this study were twofold: to analyze the prevalence and correlates of IPV in 15–49-year-old ever-married women using DHS2019 data and to analyze the trend of three IPV subtypes by ethnicity from 2013 to 2019.

## Methods

### Study area and data source

Secondary analysis using the 2019 cross-sectional Sierra Leone Demographic and Health Survey (SLDHS2019) data was conducted. The Population and Housing Census conducted in 2015 (PHS2015) was used as the sampling frame for this 2019 survey. Sierra Leone is administratively divided into provinces, which are further subdivided into 16 districts. SLDHS2019 is a nationally representative survey that also provides representative indices at the district and ethnic levels. During the census, the country was subdivided into convenient areas, referred to as the enumeration areas (EAs). The data collection phase for the SLDHS2019 lasted from May 15 to 31 August 2019. The SLDHS2019 used a two-stage cluster sample design; in the first stage, clusters (EAs) were selected, while in the second stage, households were selected. In total, 578 EAs were selected with probability proportional to the EA size, and 24 households were selected in each EA/cluster using an equal probability systematic sampling method, resulting in the selection of 13,793 households. Out of these selected households, 13,602 were occupied, and 13,399 were successfully interviewed, with a response rate of 98.5%. The 15-49-year-old women in the selected households were eligible for the SLDHS2019. The survey was administered to men in half of the selected households. In this subsample of households used for men, one woman was randomly selected in each household and also administered the domestic violence module that included questions on intimate partner violence. There were 5,322 women selected for the domestic violence module, and 5,248 (98.61%) successfully completed this module; for 74 women, privacy was not possible, and hence, they were not interviewed. While for SLDHS2013 5,334 women were selected for the domestic violence module, only 5,185 were interviewed, owing to lack of privacy, interruptions during interview, or or failure to communicate with the women in spite of several attempts.

The SLDHS2019 was implemented by Statistics Sierra Leone on behalf of the Ministry of Health. ICF International provided technical assistance for the implementation of the survey. Ethical approval for the SLDHS2019 was granted by the Sierra Leone Ethics and Scientific Review Committee and the ICF Institutional Review board. After obtaining approval from the Measure DHS, the SLDHS2013 and SLDHS2019 data were downloaded in Stata format from the Measure website www.measuredhs.com. A detailed survey methodology and the procedures for the generation of the survey weights, including questionnaires for both surveys, are provided in the country reports and are available for free download.

### Study variables

As in other DHSs conducted in over 90 countries, including African countries, for SLDHS2019 the questions pertaining to IPV were based on the modified version of the Conflict Tactics Scale, with empirically demonstrated validity and reliability in community and clinical settings (31, 32). The computation of composite IPV and three subtypes of IPV variables, including various correlates, has been previously described (20). A brief description of these computations is provided in the pertinent subsections below.

### Outcome variable

Intimate partner violence (IPV) was defined as the respondent having ever experienced either emotional, physical, and/or sexual violence from their current/former husband/partner. The IPV variable was created from several questions used in the SLDHS2019 and coded as a binary (yes/no) outcome variable. The lifetime experience of physical violence variable was created based on affirmative replies to the experiences pertaining to having ever been pushed, shaken, or had something thrown at them; being slapped; having one’s arm twisted or hair pulled; being punched with a fist or with something that could hurt; being kicked or dragged; being strangled or burned; and being threatened with a knife, gun, or any other weapon. The lifetime emotional experience variable was computed from positive answers to either having been humiliated, threatened with harm, insulted, or made to feel bad. Finally, lifetime sexual violence was computed from positive responses to either having been physically forced into unwanted sex, unwanted sexual acts, or performing unwanted sexual acts.

### Explanatory variables

Several studies using DHS data have identified IPV correlates, and in this study, 12 explanatory variables were used for association with respondents who had ever experienced intimate partner violence: the women’s age, educational attainment, and occupation, the partners’ educational attainment, the households’ wealth status, number of living children, urban/rural residency status, involvement in decision-making, IPV acceptance, alcohol use by the husband/partner, knowledge of the father beating the mother, and marital control behavior exhibited by the husband/partner.

### Statistical analysis

All analyses were conducted using STATA version 17 (Texas, United States) incorporating survey procedures to correctly account for the complex sample design characteristics of clustering, stratification, and sampling weights, using two-sided tests with a statistical significance of <0.05.

SLDHS2019 and SLDHS2013 datafiles were downloaded from the DHS website in the STATA file format. The analysis entailed computing unweighted counts, the number of missing records, and weighted percentages for the outcome and all explanatory variables; missing values were not imputed. This was followed by bivariate and multivariable analysis using the simple and multiple binary logistic regression models. All explanatory variables that were found to be statistically significantly associated with the outcome variable of IPV were added to the final multivariable logistic regression model. Odds ratios, their corresponding 95% confidence intervals, and statistical significance were computed.

Linear trend analysis of IPV prevalence between the two surveys was performed next by appending the two data files. The dichotomous variable “year”, representing the two survey years, was created and used in the binary simple logistic regression model. The IPV type was used as the explanatory variable in this logistic model. Proportions were reported as percentages while factoring the complex survey design. Linear trend analysis over the course of two DHSs was performed for nine ethnicities and three types of IPV.

## Results

Out of the 5,248 women who were selected and interviewed for the domestic violence module, 1,193 were never married. Hence, information on IPV was obtained from 4,055 women. In total, 2,428 women reported having ever experienced emotional, physical, and/or sexual violence perpetrated by an intimate partner. Emotional, physical, and sexual forms of violence were reported by 1,859, 1,941, and 311 women, respectively. Regarding the number of women reporting various groupings of IPV types, 1,388 women reported both physical and emotional IPV, 272 women reported both emotional and sexual IPV, 243 women reported both physical and sexual IPV, and 220 women reported all three types of IPV.

Table 1 presents the results of the outcome and explanatory variables in terms of unweighted counts and weighted percentages based on the 4,055 ever-married women aged 15–49 years who answered questions pertaining to IPV in the domestic violence module of SLDHS2019. For 239 women, information on decision-making in the areas of large household purchases, visits to relatives, healthcare seeking for the self, and the educational attainment of their husband/partner was unavailable since these questions were asked to women who were either currently married or living in a union with a man.

**Table 1:** Counts and proportions of study variables – Sierra Leone demographic and health survey 2019.

Variable	Unweighted count (*n* = 4,055)	Percentage (weighted)
**Outcome variable**		
Intimate partner violence (emotional, physical, and/or sexual)	Yes = 2,428	60.81 (58.48–63.10)
Emotional violence	Yes = 1,859	45.90 (43.47–48.34)
Physical violence	Yes = 1,941	49.81 (47.50–52.12)
Sexual violence	Yes = 311	8.14 (6.92–9.56)
**Explanatory variables**		
Age	15–19y = 187	4.72
	20-24y = 589	13.81%
	25–29y = 843	19.92%
	30-34y = 742	17.73%
	35-39y = 792	19.96%
	40-44y = 469	12.74%
	45-49y = 433	11.13%
Respondent’s education	No education = 2,574	60.74%
	Primary = 498	12.94%
	Secondary = 862	22.92%
	Higher = 121	3.40%
Husband’s education	No education/Unknown = 2,241	56.15%
	Primary = 317	8.18%
	Secondary = 948	26.20%
	Higher =310	9.47%
Occupation	Professional/Technical/Managerial/Clerical/ Sales/Services = 1,058	29.73%
	Not working = 586	15.62%
	Agriculture/self-employed/Skilled manual/Unskilled manual/ Other = 2,411	54.65%
Wealth	Poorest = 1,106	22.16%
	Poorer = 879	21.17%
	Middle = 831	20.47%
	Richer = 699	18.50%
	Richest = 540	17.70%
Residence	Urban = 1,263	37.05%
	Rural = 2,792	62.95%
Children	0 = 261	6.86%
	1–2 = 1,475	37.42%
	3–4 = 1,439	34.63%
	5–12 = 880	21.09%
Decision-making	Participated = 2,286	59.09%
	Not participated = 1,530	40.91%
Acceptance	Not justified/Unknown = 1902	47.25%
	Justified = 2,153	52.75%
Alcohol use	No = 3,341	82.15%
	Yes = 714	17.85%
Knowledge of father beating mother	No/Unknown = 2,951	71.72%
	Yes = 1,104	28.28%
Marital control	No/Unknown = 850	20.80%
	Yes = 3,205	79.20%

* Questions on “husband’s education” and “decision-making” were asked to currently married women only.

In women aged 15–49 years, the lifetime prevalence of having ever experienced IPV (emotional, physical, and/or sexual) committed by either their husband or partner was 60.81% (95% CI: 58.48–63.10).

Physical IPV was the most common form of IPV reported (49.81%: 95%CI = 47.50–52.12), closely followed by emotional IPV (45.90%: 95% CI = 43.47–48.34), while sexual IPV was the least common type of reported IPV (8.14%: 95% CI = 6.92–9.56). Regarding the most common form of IPV reported within each of the three IPV types, the most common physical, emotional, and sexual IPV forms were having ever been slapped (45.53%: 95%C1 = 43.12–47.96), insulted or made to feel bad (40.15%: 95%CI = 37.81–42.53), and forced into unwanted sex (6.29%: 95%CI = 5.27–7.48), respectively.

Over half (56.18%) of the respondents were under 35 years old; most (60.74%) had no formal education, and similarly, 56.15% of the women reported that their husband/partner either had no formal education or they did not know about their educational attainment; 15.62% did not work, but over half (54.65%) were engaged in agriculture, unskilled/skilled manual labor, or were self-employed. Whereas 43.33% of the women hailed from households comprising of the poorest and poorer groups of the wealth index, 62.95% were rural dwellers; 72.05% had one to four living children. Most (59.09%) respondents participated in decision-making when it came to decisions entailing the respondent’s healthcare, large household purchases, and/or visits to family/relatives; these decisions were either made alone or with their husbands. Over half (52.75%) believed that beating by the husband was justified, i.e., acceptable if the wife goes out without telling her husband, neglecting children, arguing, refusing sex, and/or burning food. Alcohol use by the husband/partner was attested to by 17.85% of the respondents; 28.28% had reported having knowledge of parental IPV in terms of the father beating the mother; and over three-quarters (79.20%) reported marital control behavior exhibited by their husband/partner.

The results of the simple and multivariable logistic regression models are presented in Table 2. Crude and adjusted odds ratios along with the corresponding 95% confidence intervals and statistical significance are provided. In all, 9 out of 12 explanatory variables were statistically significantly associated with IPV in the bivariate analysis, and the same were used for the multivariable model, i.e., the women’s age and educational attainment, partners’ educational attainment, wealth status, alcohol use by husband/partner, having knowledge of the father beating the mother, marital control, IPV acceptance, and number of living children. Out of these 9 explanatory variables, only 2, i.e., household’s wealth quantile and women’s educational attainment, were not found to be statistically significant in the final model.

**Table 2:** Crude odds ratios and adjusted odds ratios for all statistically significant associations between intimate partner violence and the selected variables – Sierra Leone demographic and health survey 2019.

Explanatory variable	Unadjusted OR	*p*-value	95% CI	Adjusted OR	*p*-value	95% CI
Age						
15–19	Reference			Reference		
20–24	1.37	0.117	0.92–2.04	1.43	0.131	0.90–2.26
25–29	1.66	0.012	1.12–2.46	1.89	0.012	1.15–3.10
30–34	1.21	0.357	0.81–1.82	1.62	0.061	0.98–2.67
35–39	0,99	0.965	0.67–1.47`	1.32	0.308	0.78–2.23
40–44	0.79	0.284	0.51–1.22	1.01	0.979	0.58–1.76
45–49	0.76	0.202	0.50–1.16`	0.99	0.966	0.55–1.76
Education (respondent/women)						
No Education	Reference			Reference		
Primary	1.60	<0.001	1.27–2.02	1.26	0.166	0.91–1.74
Secondary	1.38	0.004	1.11–1.73	1.06	0.681	0.79–1.44
Higher	0.91	0.671	0.59–1.41	0.87	0.640	0.50–1.54
Education (Husband)						
No Education	Reference			Reference		
Primary	1.22	0.196	0.90–1.66	1.07	0.726	0.74–1.54
Secondary	1.51	<0.001	1.21–1.88	1.31	0.042	1.01–1.71
Higher	1.25	0.178	0.90–173	1.05	0.818	1.70–1.57
Occupation						
Professional,	Reference			Not Applicable		
Technical, managerial, clerical, sales, services						
Not working	0.91	0.479	0.69–1.19			
Agriculture, self-employed, skilled manual, unskilled manual, other	0.93	0.518	0.74–1.16			
Wealth						
Poorest	Reference			Reference		
Poorer	1.33	0.012	1.07–1.67	1.21	0.120	0.95–1.55
Middle	1.26	0.061	0.99–1.60	1.19	0.205	0.91–1.56
Richer	1.28	0.053	0.99–1.64	1.30	0.091	0.96–1.76
Richest	1.24	0.211	0.89–1.72	1.39	0.148	0.89–1.19
Residence						
Urban	Reference			Not Applicable		
Rural	0.96	0.683	0.77–1.18			
Children						
No children	Reference			Reference		
1–2 children	1.46	0.035	1.03–2.09	1.49	0.037	1.02–2.15
3–4 children	1.08	0.676	0.75–1.55	1.20	0.379	0.80–1.79
5–12 children	1.01	0.969	0.69–1.47	1.50	0.069	0.97–2.31
Decision-making						
Did not participate	Reference			Not Applicable		
Participated	1.02	0.816	0.86–1.22			
Acceptance of IPV						
Not justified	Reference			Reference		
Justified	1.80	<0.001	1.49–2.17	1.55	<0.001	1.26–1.90
Alcohol use by husband						
Does not use alcohol	Reference			Reference		
Uses alcohol	3.82	<0.001	2.96–4.92	3.52	<0.001	2.67–4.63
Knowledge of father beating mother						
No	Reference			Reference		
Yes	2.30	<0.001	1.84–2.88	2.34	<0.001	1.85–2.95
Marital control by husband						
No	Reference			Reference		
Yes	5.47	<0.001	4.26–7.02	4.38	<0.001	3.32–5.77

OR, odds ratio, CI, confidence interval.

Results from the multivariable logistic regression model show that compared with women in the 15-19-year age group, women in the 25-29-year age group experienced an increase (aOR: 1.889; 95%CI: 1.52–3.096) in IPV. Compared with women whose husbands had no formal education, those women whose husbands had secondary education experienced an increase (aOR: 1.314; 95%CI: 1.010–1.709) in IPV. Women with 1–2 living children experienced an increase (aOR: 1.485; 95%CI: 1.025–2.151) of IPV in comparison to women who had no children. The odds of experiencing IPV were 1.546 times (95% CI: 1.257–1.901) higher in women who opined that IPV was justified compared to women who believed that it was unjustified. IPV odds were 3.517 times (95%CI: 2.670–4.634) higher in women with intimate partners using alcohol than in those whose intimate partners did not. IPV odds were 2.337 times (95%CI: 1.852–2.950) higher in women with knowledge of their father having ever beat their mother than in those who did not report such knowledge. Meanwhile, IPV odds were 4.379 times (95%CI: 3.321–5.775) higher in women whose husbands exhibited marital control than in those whose husbands did not.

Table 3 shows the proportions, 95% confidence intervals, and statistical significance of the linear trends across the two SLDHSs for emotional, physical, and sexual IPV. These indices are provided for the nine ethnicities, i.e., Fallah, Kono, Limba, Loko, Mandingo, Mende, Sherbro, Temne, and Korankoh. Regarding emotional IPV, with the exception of Sherbro, all eight other ethnicities registered an increase in prevalence from 2013 to 2019. However, this increase was statistically significant only for the Kono, Limba, Mende, Temne, and Korankoh ethnicities. Regarding physical IPV, the Fallah, Sherbro, and Korankoh ethnicities registered a decrease from 2013 to 2019. However, this decrease was not statistically significant. While the other six ethnicities reported an increase between the two surveys, only for the Kono and Temne ethnicities was this increase was statistically significant. Lastly, regarding sexual IPV, the Fallah, Kono, Limba, Loko, and Korankoh ethnicities reported an increase from 2013 to 2019. However, this increase was statistically significant only for the Fallah and Korankoh ethnicities.

**Table 3:** Proportions and trend analyses for emotional, physical, and sexual violence by ethnicity – Sierra Leone demographic and health survey (DHS) 2013 and 2019.

Type of violence		DHS 2013 percentage	95% CI	DHS 2019 percentage	95% CI	Trend statistical significance (*p*-value)
Emotional violence						
	Fallah	17.41	10.10–28.32	31.00	22.53–40.96	0.050
	Kono	15.07	11.14–20.08	52.98	45.26–60.55	<0.001
	Limba	28.71	21.49–37.20	45.23	37.53–53.18	0.005
	Loko	35.66	21.11–53.44	51.31	36.34–66.05	0.171
	Mandingo	39.95	22.19–60.81	60.66	44.05–75.12	0.121
	Mende	28.62	25.10–32.41	39.80	36.16–43.56	<0.001
	Sherbro	33.57	19.91–50.67	32.14	21.30–45.32	0.885
	Temne	33.88	29.24–38.87	51.27	46.99–55.53	<0.001
	Korankoh	18.29	11.98–26.90	45.75	36.52–55.28	<0.001
Physical violence						
	Fallah	35.37	24.96–47-38	28.87	20.97–38.31	0.364
	Kono	35.36	27.26–44.40	50.27	43.60–56.94	0.009
	Limba	49.90	41.25–58.55	50.24	42.58–57.89	0.953
	Loko	50.77	33.07–68.28	64.91	50.60–76.95	0.209
	Mandingo	45.92	27.99–64.97	52.51	38.22–66.40	0.586
	Mende	39.03	35.15–43.05	39.55	36.24–42.96	0.842
	Sherbro	36.66	24.81–50.38	31.15	18.83–46.88	0.566
	Temne	51.43	46.43–56.40	61.49	57.65–65.19	0.002
	Korankoh	45.94	35.22–57.05	45.66	37.02–54.56	0.968
Sexual violence						
	Fallah	3.73	1.66–8.15	9.65	4.86–18.26	0.070
	Kono	3.37	1.29–8.54	9.91	5.98–16.00	0.043
	Limba	7.42	3.83–13.92	11.11	6.21–19.11	0.353
	Loko	5.29	2.16–12.37	7.79	2.77–19.99	0.554
	Mandingo	12.73	2.94–41.23	12.27	5.76–24.26	0.962
	Mende	4.15	2.92–5.87	3.78	2.61–5.43	0.713
	Sherbro	8.14	3.11–19.69	5.45	1.68–16.24	0.579
	Temne	10.42	7.93–13.58	9.71	7.54–12.42	0.703
	Korankoh	8.73	4.17–17.38	19.28	12.58–28.40	0.049

CI, confidence interval.

## Discussion

Intimacy with men hurts some women, and Sierra Leone is no exception to this global rule. This study examined the lifetime prevalence and correlates of IPV victimization in women by conducting secondary analysis of the nationally and ethnicity representative SLDHS2019 cross-sectional data. The trends of emotional, physical, and sexual IPV by ethnicity were also examined between the SLDHS2013 and SLDHS2019. The lifetime IPV prevalence among ever-married Sierra Leonese women aged 15–49 was a staggering 60.81%. The intimate partner was defined as either the most recent husband/partner for the divorced, separated, or widowed women or the current husband/partner for the currently married women. The prevalence of having ever experienced sexual and/or physical IPV was 51.67% (95% CI: 49.24–54.08); hence, over half of women had experienced these two types of IPV in their lifetime, either exclusively or in conjunction with the other. There is a contrast with the global and WHO Africa Region estimates of physical and/or sexual IPV in a similar demographic, which in 2018 were 27 and 33%, respectively (3); the victimization and resultant harm among Sierra Leonean women exceeded the global average by 24.67 percentage points and that of the WHO Africa Region by 18.67 percentage points. Among the three types of IPV, the lifetime prevalence of physical IPV was the highest, which is a finding in consonance with other studies (20, 26).

Association of IPV was examined individually for the 12 explanatory variables, out of which 9 were found to be statistically significant. Occupational status, residential status, and decision-making participation were not statistically significantly associated with IPV. A recent study from Gambia also reported a similar lack of statistically significant association with IPV via bivariate analysis using the DHS data (26), contrasting with the recent meta-analysis that reported the association of residency in rural areas with IPV (5).

In the final multivariable model that included nine statistically significant explanatory variables found in the bivariate analysis, the highest adjusted odds (aOR: 4.38) of experiencing IPV were associated with the marital control shown by the respondents’ intimate partner, followed by alcohol use by the husband/partner (aOR: 3.52); meanwhile, the third highest explanatory variable was the respondent having knowledge of her father beating her mother (aOR: 2.34). Marital control or controlling behavior exhibited by men toward women has been consistently reported to be a strong correlate of the IPV experience of women (10, 26, 29). Controlling behavior perhaps reveals an antecedent pattern that results in violence when the perpetrator perceives loss or compromised control in an intimate relationship context. The association of alcohol use by the male partner has been another consistent finding in the context of IPV experienced by women (9, 11, 17, 18, 20, 22, 24, 26). Loss or compromised impulse control and inhibitions is a common effect of alcohol and perhaps mediates IPV though this mechanism. However, DHS surveys do not inquire about the frequency and quantity of alcohol consumed by the male partner; hence, dose–response associations could not be elucidated. Nonetheless, mere reported use of alcohol by the man partner and its strong and consistent association with IPV is staggering, making it important when crafting IPV prevention policies and addressing practices pertaining to health education and promotion activities. Yet a few studies do not report such strong associations between alcohol use by the male partner and the IPV experiences of women (21, 26). One such study was from Afghanistan, where alcohol consumption is rather rare (21), while another study from Gambia reported only 38 respondents who reported use of alcohol by their male partners. Women reporting having knowledge of their father beating their mother, and its association with their experiencing higher prevalence of IPV, is another disturbing but consistent finding reported in several studies (14, 20–22, 26). A mediating mechanism might entail the normalization and acceptance of IPV by women as a learned behavior. Ostensibly, men, while growing up, also learn about such harmful behavior from their fathers and accept it as a norm in their intimate relationships as well.

The acceptance of IPV by women is another learned behavior that was statistically significantly associated with IPV perpetrated by men in Sierra Leone and has been reported by several other studies (14, 15, 20, 21). Is IPV acceptance on the part of women somehow a self-fulfilling prophecy? The question could not be answered in the context of a cross-sectional study as both accepting attitude and IPV experience are elucidated at the same time as opposed to temporally. This is a major limitation of studying complex phenomena such as IPV in cross-sectional studies as only associations could be expounded. The association of higher odds of IPV with the number of living children showed statistical significance for having one to two children compared with no children in this study. However, there has been a mixed empirical relationship reported with the number of children. One study reported higher IPV prevalence among infertile women (8), while other studies have reported paradoxical effects, with higher odds of IPV in women with three or more children (9, 18), protective effects in having one to two children (20), and no effects on IPV considering the number of living children (26). Concerning the age of the respondents, the only statistical significance was found between the age group of 25–29 years versus 15–19 years old and IPV in this study. While age was not reportedly significantly associated with IPV in other studies (20, 26), a higher odds of IPV among women aged 25–29 and 40–44 years old has been reported (9). The complex relationship of age with IPV underscores the need for better studying country-specific cultural mores; perhaps, in some countries, widespread IPV practices cloud this relationship.

The status of households in terms of wealth and the educational attainment of both the women and their intimate partner were not statistically significant in the final multivariable logistic regression model in this study. More pervasive IPV practices perhaps negate the effects of these factors’ association with IPV in this study. However, several studies have reported an association of higher wealth status, measured in quintiles ranging from poorest to richest, and its protective effects on women experiencing IPV (15, 16, 23, 26) with higher odds of IPV in women hailing from lower wealth quintiles (17, 25). The lack of statistically significant association with the educational attainment of the respondent or her intimate partner was another unusual finding from this study. Several studies have reported an association of IPV with women’s low educational attainment and that of their partners (5, 13, 18, 22); conversely, higher educational attainment was found to serve as a protective factor against IPV (5, 19, 21, 23, 26). Even parental educational attainment of less than high school has been associated with IPV (7). IPV victims are more likely to have pre-term deliveries and low-birth-weight babies; both long-term physical and mental health are impacted in women experiencing IPV, including, adversely, their children (12, 33–38).

The national/country-level IPV indices mask the subnational disparities (5, 9, 21, 22, 26). In this study, IPV trend analysis was conducted using the SLDHS 2013 and 2019 data. The trend analysis was conducted according to ethnicity, as the administrative geographic boundaries of districts between the two survey periods were redrawn in Sierra Leone. Some ethnicities were reportedly more likely to indulge in IPV than others in Sierra Leone, underscoring the fact that in some ethnic groups, love hurts women more often than others.

In several ethnicities, the three types of IPV increased, with emotional violence registering a statistically significant increase in five out of nine ethnicities, physical IPV increasing in two ethnicities, and sexual IPV increasing in two ethnicities as well. None of the ethnicities were found to have undergone a statistically significant decrease. It is difficult to conjecture about this reported increase in IPV. Perhaps during the intervening period of six years between the two surveys, women were somewhat more initially reticent about disclosing their IPV experiences out of shame or fear, but by 2019, they became more outspoken; or, perhaps, IPV did increase during the two surveys. Nonetheless, the results highlight the role and specificity of targeting ethnicities to address and remedy this health and human rights menace.

Hence, several factors collectively conspire to perpetrate and perpetuate IPV against women, including the educational attainment of both women and their intimate partners, household wealth, the number of children, alcohol use, and culturally defined norms, practices, attitudes, beliefs, and power dynamics in the context of intimacy. Thus, remedial measures need to factor in the complex interplay of these factors, including targeted interventions by ethnicity, in Sierra Leone. Health education and promotion campaigns targeting IPV by reshaping the societal attitudes that perpetuate intimate violence are necessary and important. Moreover, a recent study based on an extensive literature review concluded that addressing IPV through legal interventions in terms of the enactment and enforcement of laws protecting women and providing relief would be paramount (39).

The strengths of this study include the use of nationally representative survey data and of a validated questionnaire, thus allowing comparisons across and between countries over the different survey years.

Meanwhile, limitations arose from the cross-sectional study design, thus only permitting the elucidation of associations. Since the respondents included ever-married women and only those between the ages of 15 and 49, the estimates and trends are not a true reflection but probably an underestimate of the IPV burden in Sierra Leone. The data collection methodology entailed self-reported answers, which are fraught with recall bias, in addition to the cultural sensitivities and feelings of shame or guilt clouding the responses.

## Conclusion

Love and intimacy with men should not hurt women. The lifetime IPV prevalence among ever-married Sierra Leonean women aged 15–49 was an extremely high 60.81%. Over half of women reported IPV, in stark contrast with the global and WHO Africa Region estimates of 27% and 33%, respectively. In the multivariable context, the strongest associations were found between IPV and marital control, having knowledge of the father beating the mother, and alcohol use by the husband/partner. Several ethnicities registered statistically significant increases in the IPV sub-types over the course of six years between the two surveys. The results highlight the complex web of factors underpinning IPV but also advocate for directing ethnicity-based remedial measures to stem and reverse the tide of IPV. To effectively address IPV in Sierra Leone, short-term measures in general should include health education and promotion activities that are specifically directed toward certain ethnicities. Meanwhile, long-term measures would entail the higher educational attainment of both men and women, including revisiting the cultural norms that confine women into the detrimental status of second-class citizens, a gross violation of their health and human rights. Future studies entailing similar analyses using data from similar surveys including the DHS would help gauge the progress of such endeavors. Such nationally representative surveys in the future would also need to expand their inclusion criteria by adding women who were never married but have or had intimate relationships with men, i.e., the major limitation of data used in this study.

## Data availability statement

The datasets presented in this study can be found in online repositories. The names of the repository/repositories and accession number(s) can be found in the article/supplementary material.

## Ethics statement

Ethical approval for the SLDHS2019 was granted by the Sierra Leone Ethics and Scientific Review Committee as well as the ICF Institutional Review board. The approval for secondary analysis of both SLDHS 2013 and SLDHS2019 was granted to the study author by the Measure DHS. The studies were conducted in accordance with the local legislation and institutional requirements. DHS survey methodology, verbal consent is obtained for participation in this study was provided by the participants’ legal guardians/next of kin.

## Author contributions

The author confirms being the sole contributor of this work and has approved it for publication.

## Conflict of interest

The author declares that the research was conducted in the absence of any commercial or financial relationships that could be construed as a potential conflict of interest.

## Publisher’s note

All claims expressed in this article are solely those of the authors and do not necessarily represent those of their affiliated organizations, or those of the publisher, the editors and the reviewers. Any product that may be evaluated in this article, or claim that may be made by its manufacturer, is not guaranteed or endorsed by the publisher.
